# Fatal Case of Israeli Spotted Fever after Mediterranean Cruise

**DOI:** 10.3201/eid1412.070641

**Published:** 2008-12

**Authors:** Noémie Boillat, Blaise Genton, Valérie D’Acremont, Didier Raoult, Gilbert Greub

**Affiliations:** University Hospital of Lausanne, Lausanne, Switzerland (N. Boillat, B. Genton, V. D’Acremont, G. Greub); Mediterranean University, Marseilles, France (D. Raoult)

**Keywords:** Obligate intracellular bacteria, *Rickettsia conorii* subsp. *israelensis*, spotted fever group rickettsia, mortality, travel, *Rickettsiales*, Israeli spotted fever, Mediterranean spotted fever, letter

**To the Editor:** Israeli spotted fever (ISF) is caused by *Rickettsia conorii* subsp. *israelensis*. This recently described subspecies is genetically close to *R. conorii* subsp. *conorii*, the agent of Mediterranean spotted fever (MSF) (*1*,*2*). ISF is likely transmitted by the dog tick *Rhipicephalus sanguineus* (*3*). This tick, which is also the vector of *R. conorii* subsp. *conorii*, has low affinity for hosts other than dogs. Therefore, like MSF, cases of ISF will likely be sporadic (*4*,*5*). ISF was first reported in Israel (*1*) and was also recently described in Portugal and Italy (*6*–*8*). The clinical manifestations of ISF are similar to those of other spotted fever group infections, but an inoculation eschar is rarely observed and a history of tick exposure is not always present (*4*–*6*,*9*). The incubation period is ≈7–8 days after the tick bite (*4*).

We describe a 63-year-old man who had fatal ISF despite adequate therapy. The patient, who lived in Switzerland, took a cruise on the Mediterranean Sea, sailing for a week along the coasts of Crete, Libya, and Malta (Figure). With his wife, he visited several archeological sites in Libya (Cyrene, Apollonia, Ptolemais, Leptis Magna, Sabratha). Three days after returning to Switzerland, the patient reported loss of appetite, epigastric pain, and loose stools. Four days later, a fever (40°C) and generalized rash developed. The patient was hospitalized 6 days after symptom onset. At that time, he had fever (38.3°C), hypotension (85/55 mm Hg), tachycardia (100/min); a maculopapular rash involving the trunk, limbs, palms, and soles; and petechial lesions on the right arm. The patient was confused and exhibited bilateral dysdiadochokinesis. Laboratory investigations yielded the following results: C-reactive protein level 183 mg/L; leukocyte count 4.9 ×10^9^/L; platelet count 23 ×10^9^/L; creatinine 741 μmol/L; sodium 127 mmol/L; aspartate aminotransferase 299 U/L; alanine aminotransferase 156 U/L; γ-glutamyl transpeptidase 160 U/L; pH 7.45; and lactate 4.5 mmol/L. A rapid blood test for malaria (OptiMAL-IT, DiaMed, Cressier, Switzerland) had negative results, and peripheral blood smears did not show any *Plasmodium* spp. During his trip, the patient had not had contact with animals and had no history of tick bite. His wife was asymptomatic.

**Figure:**
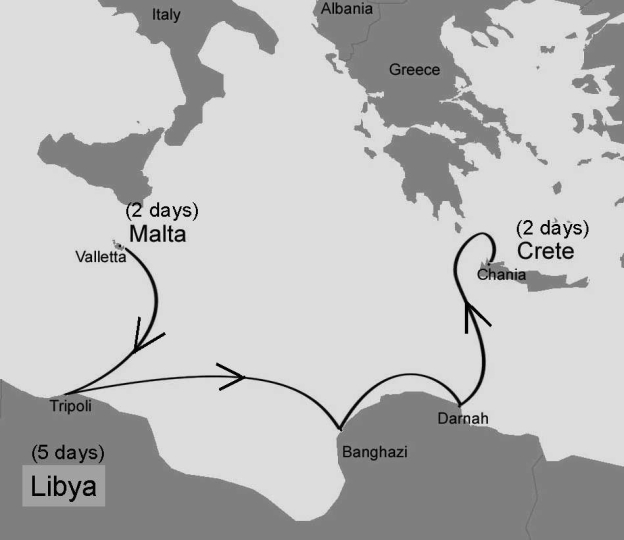
Cruise path on the Mediterranean Sea along the coasts of Crete, Libya, and Malta.

The initial differential diagnosis mainly included typhoid fever, leptospirosis, malaria, HIV seroconversion, and MSF. Treatment with intravenous doxycycline, 100 mg twice a day, and ceftriaxone, 2 g every 24 h, was immediately started. The patient was admitted to the intensive care unit because of hypotension and received vasopressors. Twenty-four hours later, renal function deteriorated, and the patient was transferred to our university hospital in Lausanne, Switzerland, for hemodialysis. Antimicrobial drug treatment was switched to intravenous imipenem, 500 mg 4 times a day, and clarithromycin, 500 mg twice a day. On day 8 after symptom onset, he had pulseless electrical heart activity probably caused by metabolic acidosis. He died on day 11 after symptom onset.

Blood was sterile but was not cultured for rickettsiae, and stool culture showed no pathogens. Using serum obtained on day 6, we conducted a *Rickettsia* spp. microimmunofluorescence test; results were negative. However, results of a skin biopsy, conducted after 2 days of antibiotherapy, were positive by 2 PCRs targeting the *omp*A and *glt*A genes (*10*). Sequencing allowed postmortem identification of the etiologic agent as *R. conorii* subsp. *israelensis.* The sequences of *omp*A exhibited 99.8% (532/533 bp) similarity with *R. conorii* subsp. *israelensis* strain ISTT-CDC1^T^ (GenBank accession no. U43797), 98.3% (524/533 bp) with *R. conorii* subsp. *caspia* strain A-167^T^ (U43791) and 96.8% (540/558 bp) with *R. conorii* subsp. *conorii* strain Malish^T^ (AE008674). The sequences of *glt*A exhibited 100% (177/177 bp) similarity with *R. conorii* subsp. *israelensis* (U59727), 99.4% (176/177 bp) with *R. conorii* subsp. *caspia* (U59728), and 98.3% (174/177 bp) with *R. conorii* subsp. *conorii* (AE008677).

Autopsy showed severe pulmonary edema and liver ischemia. The brain had petechial hemorrhages in the left cerebellum and right frontal lobe, as well as a small recent ischemic infarction in the right caudate nucleus.

In this case of ISF, delayed medical consultation and thus late initiation of antimicrobial drug therapy (6 days after symptoms onset), the patient’s age, and his chronic alcohol abuse probably contributed to the fatal course. The virulence of *R. conorii* subsp. *israelensis* might also be higher than that of *R. conorii* subsp. *conorii* (*6*,*7*).

ISF has not been described in any of the countries visited by the patient. Given the incubation time of spotted fever (7–8 days), he was probably infected in Lybia, where he spent days 6–10 before symptom onset. Geographic distribution of ISF can thus be extended to North Africa. Since ISF and MSF share the same vector (*Rh. sanguineus)*, disease-endemic areas probably overlap.

This report also points out the importance of early empirical treatment. Rickettsiosis should be suspected in febrile travelers, especially when they have a rash, even in the absence of history of tick exposure and inoculation eschar. Counseling before travel to areas endemic for spotted fever rickettsioses should include preventive measures for tick bites and recommendations to immediately seek medical advice in case of fever.

## References

[1] Zhu Y, Fournier PE, Eremeeva M, Raoult D. Proposal to create subspecies of *Rickettsia conorii* based on multi-locus sequence typing and an emended description of *Rickettsia conorii.* BMC Microbiol. 2005;5:11. 10.1186/1471-2180-5-11 15766388 PMC1079849

[2] Fournier PE, Dumler JS, Greub G, Zhang J, Wu Y, Raoult D. Gene sequence-based criteria for identification of new *Rickettsia* isolates and description of *Rickettsia heilongjiangensis* sp. nov. J Clin Microbiol. 2003;41:5456–65. 10.1128/JCM.41.12.5456-5465.2003 14662925 PMC308961

[3] Giammanco GM, Mansueto S, Ammatuna P, Vitale G. Israeli spotted fever *Rickettsia* in Sicilian *Rhipicephalus sanguineus* ticks. Emerg Infect Dis. 2003;9:892–3. 12899144 10.3201/eid0907.030109 PMC3023446

[4] Gross EM, Yagupsky P. Israeli spotted fever in children. A review of 54 cases. Acta Trop. 1987;44:91–6. 2884843

[5] Wolach B, Franco S, Bogger-Goren S, Drucker M, Goldwasser RA, Sadan N, Clinical and laboratory findings of spotted fever in Israeli children. Pediatr Infect Dis. 1989;8:152–5. 2710586

[6] Bacellar F, Beati L, França A, Poças J, Regnery R, Filipe A. Israeli spotted fever rickettsia (*Rickettsia conorii* complex) associated with human disease in Portugal. Emerg Infect Dis. 1999;5:835–6. 10603225 10.3201/eid0506.990620 PMC2640813

[7] Giammanco GM, Vitale G, Mansueto S, Capra G, Caleca MP, Ammatuna P. Presence of *Rickettsia conorii* subsp. *israelensis*, the causative agent of Israeli spotted fever, in Sicily, Italy, ascertained in a retrospective study. J Clin Microbiol. 2005;43:6027–31. 10.1128/JCM.43.12.6027-6031.2005 16333093 PMC1317185

[8] De Sousa R, Ismail N, Doria-Nobrega S, Costa P, Abreu T, França A, The presence of eschars, but not greater severity, in Portuguese patients infected with Israeli spotted fever. Ann N Y Acad Sci. 2005;1063:197–202. 10.1196/annals.1355.032 16481514

[9] Yagupsky P, Wolach B. Fatal Israeli spotted fever in children. Clin Infect Dis. 1993;17:850–3. 8286624 10.1093/clinids/17.5.850

[10] Fournier PE, Raoult D. Suicide PCR on skin biopsy specimens for diagnosis of rickettsioses. J Clin Microbiol. 2004;42:3428–34. 10.1128/JCM.42.8.3428-3434.2004 15297478 PMC497613

